# Annual exposure to PM_10_ is related to cerebral small vessel disease in general adult population

**DOI:** 10.1038/s41598-022-24326-y

**Published:** 2022-11-16

**Authors:** Han-Yeong Jeong, Hyun-Jin Kim, Ki-Woong Nam, Su-Min Jeong, Hyuktae Kwon, Jin-Ho Park, Hyung-Min Kwon

**Affiliations:** 1 Department of Neurology, Emergency Medical Center, Seoul National University Hospital, Seoul, Republic of Korea; 2 Seoul National University College of Medicine, Seoul, Republic of Korea; 3 National Cancer Control Institute, National Cancer Center, Goyang, Republic of Korea; 4 Department of Neurology, Seoul Metropolitan Government-Seoul National University Boramae Medical Center, Seoul National University College of Medicine, 20 Boramae-Ro 5-Gil, Dongjak-Gu, Seoul, 07061 Republic of Korea; 5 Department of Family Medicine, Seoul National University Hospital, Seoul National University College of Medicine, 101 Daehak-Ro, Jongro-Gu, Seoul, 03080 Republic of Korea

**Keywords:** Environmental sciences, Neurology, Risk factors

## Abstract

Ambient air pollution is one of the most important global health issues. Although several studies have been reported the associations between air pollution and brain function or structure, impact of the air pollution on cerebral small vessel disease (cSVD) have rarely been explored in Asian adult population. We evaluated the association between exposure to air pollutants and cSVD in Korean asymptomatic adults. This cross-sectional study included 3257 participants of a health screening program from January 2006 to December 2013. All participants performed brain magnetic resonance imaging. To assess the cSVD, we considered three features such as white matter hyperintensities (WMH), silent lacunar infarction (SLI), and cerebral microbleeds (CMBs). The annual average exposure to air pollutants [particulate matter ≤ 10 μm in aerodynamic diameter (PM_10_), nitrogen dioxide (NO_2_), sulfur dioxide (SO_2_), and carbon monoxide (CO)] was generated. The mean [standard deviation (SD)] age of the total 3257 participants was 56.5 (9.5) years, and 54.0% of them were male. Among all the included participants, 273 (8.4%) had SLI and 135 (4.1%) had CMBs. The mean volume (± SD) of WMH was 2.72 ± 6.57 mL. In result of linear regression analysis, the volume of WMH was associated with various potential factors including age, height, weight, smoking and alcohol consumption status, blood pressure (BP), hypertension, and diabetes mellitus. SLI-positive group, compared to the SLI-negative group, was older, shorter, and had higher BP as well as higher frequency of hypertension and diabetes mellitus. After adjusting for covariates, the annual average concentration of PM_10_ was significantly associated with the volume of WMH [*β* (95% CI) for Model 1 = 0.082 (0.038- 0.125), *p* < 0.001; *β* (95% CI) for Model 2 = 0.060 (0.013, 0.107), *p* = 0.013]. CMBs were not associated with the annual average concentration of PM_10_. No significant associations of NO_2_, SO_2_, and CO with cSVD were observed. In conclusion, PM_10_ exposure is associated with significant increases in brain WMH’ volume and silent lacunar infarcts in asymptomatic adults.

## Introduction

Air pollution is recognized as a major health concern leading to an increased risk of several diseases and deaths^1^. Recent studies have shown that exposure to air pollutants, especially particulate matter (PM), has detrimental effects on stroke and myocardial infarction^2–4^. PM is classified into two types, PM_10_ (diameter of 10 µm or less) and PM_2.5_ (diameter of 2.5 µm or less), according to the particle size. The size of these particles depends mainly on the source of their origin including industrial emissions and combustion of fossil fuel^2^. In fact, compared to PM_10_, PM_2.5_ exposure has been considered as a stronger culprit of the negative effects of air pollution on cardiovascular disease^5^.

Most epidemiological studies on air pollution and cardiovascular diseases have focused on short-term exposure to air pollutants and their effects on mortality or morbidity. In a previous study from Israel, associations between ischemic stroke and increased concentrations of PM_10_ or PM_2.5_ were observed among young adults aged under 55 years^6^. This association was stronger in participants who lived close to a main road. In another study from eight Chinese cities, increased concentrations of air pollutants such as PM_10_, nitrogen dioxide (NO_2_), and sulfur dioxide (SO_2_) were associated with daily stroke mortality^7^. In a study based on the stroke registry database, short-term exposure to air pollutants was associated with cardioembolic stroke^8^.

With respect to cerebrovascular disease, several studies have reported the associations with air pollution exposure, but most of them are limited to the specific group such as children or women or the occurrence of cardiovascular events^9–17^. Lilian and colleagues (2011) found that exposure to air pollution is closely related to brain volume and cognition in children^13^. They also investigated the patterns of inflammatory biomarkers, magnetic resonance imaging (MRI) volume growth, white matter hyperintensities (WMH), and cognition in children from the urban cohorts exposed to air pollution, and demonstrated the effect of a modulation of inflammatory cytokines on air pollution-associated brain volumetric responses and cognition^15^. In 2015, a women's health initiative memory study reported the negative effects of PM_2.5_ on brain white matter volume in older women^16^. Furthermore, exposure to air pollution has been well known as a potential contributor to cognitive decline and dementia^17^. In addition, several epidemiologic studies have reported on the association between long-term exposure to air pollution and cerebrovascular disease^9–12^. In 2013, a meta-analysis reported that exposure to PM_2.5_ was associated with a pooled excess risk of 11% for cardiovascular mortality for every increment of 10 μg/m^3^^18^. However, these studies focused on the occurrence of cardiovascular events, and did not adjust for traditional risk factors for cardiovascular disease such as hypertension, diabetes, and smoking. As most of the existing literature is based on linkage of mortality or administrative data such as insurance claim, previous studies had a limited ability to assess the impact of air pollution on normal individuals.

Cerebral small vessel disease (cSVD) refers to the syndrome of clinical, cognitive, neuroimaging, and neuropathological findings that affects the small arteries, arterioles, capillaries, and venules of the brain^19^. The main features of cSVD on MRI include WMH, silent lacunar infarction (SLI), cerebral microbleeds (CMBs), and perivascular spaces^20^. The cSVD is considered as a predictor of many clinical outcomes including stroke and cognitive impairment^21^. Therefore, it is important to identify the factors affecting cSVD. However, few studies have assessed the relationship between long-term air pollution and cSVD in Asian adult population. We evaluated this association, hypothesizing that the prevalence of asymptomatic cSVD would be greater among general adults to higher levels of ambient air pollutants.

## Methods

### Study population

The study subjects were participants of a health screening program conducted at the Seoul National University Hospital Health Promotion Center from January 2006 to December 2013. In South Korea, a government organization named NHIS (National Health Insurance Service) provides general health check-up services with free of charge to all Korean adults every one or two years. Therefore, general Korean adults are very accustomed to taking comprehensive periodic health screening program for disease prevention and early detection irrespective of their current health status. However, many people feel that service items provided by NHIS is not detailed and enough for their health status evaluation. Fortunately to the people, medical examination fee using high technology devices such as CT, MRI and endoscopy is very low compared to other developed countries like the US. Therefore, many people tend to take regular private health examination with their own cost at the specialized centers usually runed by big university hospitals. Almost all big hospitals in Korea run large, modernized health check-up centers equipped with high technology medical devices such as multi-dimensional CT, high resolution MRI, endoscope et al. The people choose proper program according to their specific health concerns and recommendation from their physician or a counselling nurse. In case of brain MRI/MRA, due to the high cost and characteristics of brain diseases (late onset), the participants tend to be relatively older and economically wealthier than other program recipients. In case of neurologically symptomatic people, they tend to visit a neurologist directly due to prompt early intervention with insurance coverage. In our center, people who want to take intensive health screening test including brain MRI should wait for several months with no health insurance. Therefore, the participants who took brain MRI at our center are general adults with relatively higher mean age and income status than other basic program participants. We excluded participants who had history of stroke or severe neurological deficit (*n* = 54), and malignancies (*n* = 90). Finally, a total of 3257 asymptomatic participants were recruited. They had not experienced stroke or transient ischemic attack, as mentioned in a self-completed questionnaire, and had no symptoms or signs of neurological manifestations, such as motor or sensory deficits at physical examination. Clinical information of the participants, anthropometric factors, medications, smoking, and alcohol consumption statuses were obtained using structured questionnaires, and face-to-face interviews. All participants provided informed consent, and the study was approved by the Institutional Review Board of Seoul National University Hospital (IRB No. 1708-017-873). All methods were performed in accordance with the Declaration of Helsinki and relevant guidelines and regulations.

### Diagnosis of cerebral small vessel disease

CSVD was classified as SLI, CMBs, and WHM. The presence of SLI, CMBs, and the volume of WMH were assessed through axial MRI. All participants were examined at a field strength of 1.5 T (Signa; GE Healthcare, Milwaukee, WI, USA or Magnetom SONATA; Siemens, Munich, Germany). The imaging protocol consisted of T2*-weighted gradient echo (repetition time/echo time 824/25 ms; flip angle 20°; and matrix size 320 × 179), T1-weighted spinecho (repetition time/echo time 500/11 ms), and fluid-attenuated inversion recovery (FLAIR) (repetition time/echo time 8,800/127 ms; inversion time 2,250 ms) sequences. Images were obtained as 26 transaxial slices per scan. The slice thickness was 5 mm, with 1 mm interslice gap. SLI was defined as a focal infarction measuring 3–15 mm in diameter in the territory of perforating branches to the basal ganglia, thalamus, internal capsule, corona radiata, centrum semiovale, brainstem, or cerebellum^20^. CMBs were defined as well-defined focal areas of dark signal intensity measuring ≤ 10 mm in diameter, with blooming artefacts on gradient echo MRI^20^. The volume of WMH was assessed on the axial fluid-attenuated inversion recovery sequence images. Scans were converted from the DICOM to analyse the format, using MRIcro software (University of Nottingham School of Psychology, Nottingham, UK; http://www.mricro.com), for computer-assisted determination of WMH volume^22^. The WMH volumes were measured semi-automatically and quantitatively using the MRIcro format.

### Assessment of air pollution exposure

We obtained the monitoring data from the Ministry of Environment of Korea (https://www.airkorea.or.kr). The data of 24-h concentrations of ambient air pollutants [(PM_10_, NO_2_, SO_2_, and carbon monoxide (CO)] were collected. These data were measured at more than 300 nationwide monitoring stations in South Korea. The annual average concentration of ambient air pollutant for each individual was estimated by matching the average annual concentration value corresponding to the year of the health examination visit of each subject. The zip codes of the study participants were used to match the average concentration of ambient air pollutants at the nearest monitoring stations.

### Statistical analyses

Data were summarized as the mean ± SD for continuous variables or the number of participants (percentage) for categorical variables. We assessed the associations between independent variables and each cSVD markers. The comparisons across the groups with a binary outcome such as SLI or CMB was examined using simple logistic regression analysis. In the case of WMH volume as a continuous outcome, univariate analysis was performed through simple linear regression analysis. In addition, to evaluate the difference in exposure of air pollutants between the groups with and without cSVD, we performed student’s t-test. Correlation coefficients between air pollutants were estimated using Pearson’s correlational analysis. Multiple linear regression analyses were performed to evaluate the association between each air pollutant and the volume of WMH. Multiple logistic regression analyses were adopted to evaluate the association between each air pollutant and SLI or CMBs. With respect to multicollinearity, blood pressure (BP) as a covariate was excluded from the multiple regression analysis due to collinearity with hypertension, and the other confounders did not show significant multicollinearity (Supplementary Table 1). Multivariable models were analyzed with model 1 adjusted for patient demographic factors [age, sex, and body mass index (BMI)] and model 2 adjusted for Model 1 plus comorbidities (hypertension, diabetes mellitus, and dyslipidaemia). The estimates of air pollutants were converted by scale of the interquartile range (IQR) for each pollutant. The analyses were conducted using IBM SPSS version 22.0 (IBM Corp., Armonk, NY, USA). To evaluate statistical significance of the associations, we applied both the nominal threshold value (*p* < 0.05).

### Ethics approval and consent to participate

All participants provided informed consent, and the study was approved by the Institutional Review Board of Seoul National University Hospital (IRB No. 1708-017-873).

## Results

### Participant characteristics

The detailed characteristics of the 3,257 participants are summarized in Table 1. The mean age of the included participants was 56.5 ± 9.5 years, and 54.0% of them were male. Among all the included participants, 273 (8.4%) participants had SLI and 135 (4.1%) had CMBs; 35 (1.1%) had both lesions. The mean volume of WMH was 2.72 ± 6.57 mL. The associations of independent variables with each cSVD markers are shown in Supplementary Table 2. In linear regression analysis, the volume of WMH was associated with age, height, weight, smoking and alcohol consumption status, systolic and diastolic BP, hypertension, and diabetes mellitus. Compared with the SLI-negative group, the SLI-positive group was older, shorter, and had higher systolic and diastolic BP as well as higher frequency of hypertension and diabetes mellitus. Similar to the SLI-positive group, the CMB-positive group was also older, and had higher levels of systolic BP and higher frequency of hypertension, compared to CMB-negative group. We also evaluated the difference in exposure of air pollutants between the groups with and without cSVD (Supplementary Table 3). The number of subjects with and without cSVD was 2,669 and 588, respectively. The significant difference between the two groups was only observed in PM_10_ exposure (*p* < 0.001). The mean value of PM_10_ in group with cSVD (49.4) was higher than that of group without cSVD (48.0).

**Table 1:** Basal characteristics of study participants.

Characteristics	*n *= 3257
Age, y	56.5 (9.5)
Sex, male	1758 (54.0)
Height, cm	162.9 (8.8)
Weight, kg	64.4 (11.0)
Body mass index, kg/m^2^	24.1 (3.1)
**Smoking**	
Never	1802 (55.3)
Former-smokers	878 (27.0)
Current-smokers	577 (17.7)
**Alcohol drinking**	
Never	1433 (44.0)
Former-drinkers	246 (7.6)
Current-drinkers	1578 (48.4)
**Blood pressure**	
Systolic blood pressure, mmHg	126.1 (15.7)
Diastolic blood pressure, mmHg	76.0 (10.7)
Hypertension	1257 (38.6)
Diabetes mellitus	501 (15.4)
Dyslipidemia	702 (21.6)
**Cerebral small vessel disease**	
Volume of white matter hyperintensity, ml	2.72 (6.57)
Presence of silent lacunar infarction	273 (8.4)
Presence of cerebral microbleeds	135 (4.1)

Data are presented as mean (standard deviation) for continuous variables, or n (%) for categorical variables.

### Measurement of air pollutants

According to the Annual Report of Air quality in Korea, the annual averages of most air pollutants during the study period gradually decreased, as the years increased (Supplementary Table 4). The median distance between the patient's residential area (zip code) and the air pollutant monitoring site was 2.05 (IQR 1.17–3.59) km. The concentrations and distributions of air pollutants are shown in Supplementary Table 5. The mean concentrations of PM_10_, NO_2_, SO_2_, and CO were 49.1 μg/m^3^, 29.3 ppb, 5.2 ppb, and 0.57 ppm, respectively. The IQRs of air pollutants were 11.6 μg/m^3^ for PM_10_, 15.0 ppb for NO_2_, 1.8 ppb for SO_2_, and 0.17 ppm for CO. There was a significant positive correlation between all air pollutants.

### Association between air pollutants and volume of white matter hyperintensity

Using multiple linear regression analyses adjusted for demographics and vascular risk factors, the volume of WMH was associated with the level of PM_10_ (Table 2). In Model 1, adjusted for age, sex, and body mass index, the volume of WMH was positively associated with PM_10_ (β 0.082, 95% CI 0.038–0.125, *p* < 0.001). This significant association was also maintained in Model 2 after adjusting for other vascular risk factors (β 0.060, 95% CI 0.013–0.107, *p* = 0.013). The other air pollutants, namely NO_2_, SO_2_, and CO were not associated with the volume of WMH.

**Table 2:** Linear regression analysis results for the association between white matter hyperintensity and air pollution.

Air pollutants (IQR)^b^	Volume of white matter hyperintensity^a^								
	Unadjusted			Model 1^c^			Model 2^d^		
	*β*	95% CI	*p*	*β*	95% CI	*p*	*β*	95% CI	*p*
PM_10_ (11.6 μg/m^3^)	0.034	− 0.019, 0.087	0.205	0.082	0.038, 0.125	< 0.001	0.060	0.013, 0.107	0.013
NO_2_ (15.0 ppb)	− 0.012	− 0.061, 0.037	0.628	− 0.029	− 0.069, 0.011	0.157	− 0.026	− 0.069, 0.017	0.228
SO_2_ (1.8 ppb)	− 0.027	− 0.072, 0.019	0.248	− 0.009	− 0.046, 0.029	0.654	− 0.010	− 0.050, 0.030	0.622
CO (0.17 ppm)	0.008	− 0.038, 0.055	0.726	− 0.012	− 0.051, 0.026	0.524	− 0.005	− 0.046, 0.037	0.821

IQR, interquartile range; CI, confidence interval; PM_10_, particulate matter ≤ 10 μm in diameter; NO_2_, nitrogen dioxide; SO_2_, sulfur dioxide; CO, carbon monoxide.

^a^Volume of white matter hyperintensity was square root-transformed to achieve normality.

^b^The β coefficients and standard error in each air pollutant was scaled to the interquartile range for each pollutant, respectively (11.6 μg/m^3^ for PM_10_, 15.0 ppb for NO_2_, 1.8 ppb for SO_2_, and 0.17 ppm for CO).

^c^Model 1 was adjusted for age, sex, body mass index.

^d^Model 2 was adjusted for Model 1 plus hypertension, diabetes mellitus, and dyslipidemia.

### Association between air pollutants and presence of silent lacunar infarction or cerebral microbleeds

Multiple regression analysis for the presence of SLI and air pollutants is shown in Table 3. In Model 1, PM_10_ was significantly associated with the presence of SLI [adjusted odds ratio (aOR) 1.231, 95% CI 1.037–1.461, *p* = 0.017]. This significant association was also maintained in Model 2 (aOR 1.191, 95% CI 1.002–1.416, *p* = 0.048). The concentrations of NO_2_, SO_2_, and CO were not associated with the volume of SLI. The presence of CMBs was not significantly associated with air pollutants (Table 3).

**Table 3:** Logistic regression analysis results for the association between silent lacunar infarction, cerebral microbleeds, and air pollution.

Air pollutants (IQR)^a^	Silent lacunar infarction					
	Unadjusted		Model 1^b^		Model 2^c^	
	OR (95% CI)	*p*	OR (95% CI)	*p*	OR (95% CI)	*p*
PM_10_ (11.6 μg/m^3^)	1.134 (0.962–1.337)	0.135	1.231 (1.037–1.461)	0.017	1.191 (1.002–1.416)	0.048
NO_2_ (15.0 ppb)	1.089 (0.934–1.270)	0.274	1.047 (0.895–1.225)	0.564	1.048 (0.896–1.225)	0.562
SO_2_ (1.8 ppb)	0.951 (0.823–1.099)	0.494	0.969 (0.834–1.125)	0.677	0.963 (0.829–1.119)	0.622
CO (0.17 ppm)	1.127 (0.976–1.302)	0.104	1.109 (0.955–1.287)	0.175	1.100 (0.948–1.277)	0.210

Air pollutants (IQR)^a^	Cerebral microbleeds					
	Unadjusted		Model 1^b^		Model 2^c^	
	OR (95% CI)	*p*	OR (95% CI)	*p*	OR (95% CI)	*p*
PM_10_ (11.6 μg/m^3^)	1.059 (0.840–1.334)	0.629	1.124 (0.888–1.423)	0.330	1.075 (0.848–1.363)	0.550
NO_2_ (15.0 ppb)	0.981 (0.792–1.216)	0.864	0.953 (0.768–1.183)	0.662	0.952 (0.768–1.180)	0.655
SO_2_ (1.8 ppb)	0.863 (0.681–1.026)	0.086	0.848 (0.689–1.044)	0.120	0.842 (0.685–1.036)	0.105
CO (0.17 ppm)	0.963 (0.782–1.185)	0.722	0.949 (0.768–1.172)	0.624	0.937 (0.760–1.156)	0.545

IQR, interquartile range; OR, odds ratio; CI, confidence interval; PM_10_, particulate matter ≤ 10 μm in diameter; NO_2_, nitrogen dioxide; SO_2_, sulfur dioxide; CO, carbon monoxide.

^a^The odds ratio and 95% confidence interval in each air pollutant was scaled to the interquartile range for each pollutant, respectively (11.6 μg/m^3^ for PM_10_, 15.0 ppb for NO_2_, 1.8 ppb for SO_2_, and 0.17 ppm for CO).

^b^Model 1 was adjusted for age, sex, body mass index.

^c^Model 2 was adjusted for Model 1 plus hypertension, diabetes mellitus, and dyslipidemia.

## Discussion

We demonstrated that annual exposure to PM_10_ was associated with WMH volume and presence of SLI in Asian adults. This finding suggests the hypothesis that higher long-term exposure to air pollution may negatively impact the brain, even in asymptomatic individuals.

To the best of our knowledge, there are no published researches on the associations between long-term exposure to air pollution and cSVD in Asian adults. Some heterogeneous results on cSVD in other populations have been reported. In the Framingham Offspring Cohort, long-term exposure to PM_2.5_ was associated with higher odds of SLI, but not WMH volume^23^. In the Cardiovascular Health Study, a 10-μg/m^3^ elevation of PM_10_ was associated with vascular dementia and worsening of white matter grade (the extent of WMH scored on a 10-point scale using library templates) but not SLI^24^. A study from the Northern Manhattan showed that there is no clear association between exposure to air pollutants, and subclinical brain infarcts and WMH volume^25^. In the ARIC study, higher mean PM_2.5_ and PM_10_ exposure in all time periods were associated with smaller deep-gray matter volumes, but not other MRI markers, including cSVD^26^. These discrepancies in association results might be explained in part by differences in the type of PM as well as the average exposure concentration, duration of exposure, exposure assessment, or study design used.

We have the following major differences from these previous papers. First, our participants had an average age of 56.5 ± 9.5 years, younger than those in other studies. Since cSVD shows a strong association with age and age-associated comorbidities such as hypertension^27,28^, the association between air pollution and cSVD might have been masked by covariates such as age in other studies. Second, the concentrations of air pollutants in our results are different from those of other countries. The mean concentration of PM_10_ in our sample was 49.1 ± 8.6 μg/m^3^ and it was considerably higher when compared to those in Europe and North America^29,30^. Exposure to these higher concentrations of PM is likely to result in a significant change in cSVD. In a meta-analysis published in 2014, the relationship between risk of stroke and concentration of PM_10_ was significant in Asian countries, which have a high concentration of PM_10_ (median 71.6 μg/m^3^), whereas it was not significant in Europe and North America (median 20.6 μg/m^3^ in Europe and 23.1 μg/m^3^ in North America)^30^. Therefore, despite the relatively short exposure period, exposure to high concentrations of PM may have a strong effect on cSVD. Furthermore, the prevalence of cSVD may differ between ethnic groups, due to racial disparity. In fact, cVSD has been reported to be more prevalent in Asians compared with Europeans^31,32^ and this might be explained by ethnic differences in genetic susceptibility^31^. Lastly, we differ from previous papers in research methods such as subject recruitment, air pollution exposure assessment, and statistical analysis.

Although patients with cardiovascular disease have many risk factors such as hypertension, diabetes mellitus, and smoking, air pollution differs from other risk factors, because exposure to air pollution is unavoidable. Several mechanisms have been proposed regarding the potential for harm from air pollution. The classical inflammation hypothesis is that particles inhaled into the lungs are ingested by macrophages, and inflammatory mediators are secreted, leading to effects on the cardiovascular system^33–36^. More recently, another hypothesis suggested that nanoparticle fractions of airborne PM are able to traverse the alveolar–capillary barrier, and directly affect the vasculature and circulating blood cells^37^. In vitro study, nanoparticles activate a systemic pro-inflammatory response in cultured human cells representing systemic vascular targets^38^. Another possible mechanism is that the inhaled particles can stimulate neural sensory receptors, which triggers changes in autonomic function, leading to altered cardiovascular homeostasis^39^. Elevated airborne particulate was associated between changes in heart rate variability, such as elevated mean heart rate and decreased standard deviation of normal-to-normal intervals^39^. These mechanisms of air pollutants may influence cerebral small vasculature and cause cSVD in normal individuals.

The present study has several limitations. First, because this study is cross-sectional nature and there is no follow-up imaging data, we could not identify the exact causal relationship between air pollutants and cSVD. However, we adopted the data of air pollutants in the year of health check-up day in order to strengthen the cause-effect relationship. Second, to estimate the level of exposure to air pollutants in the areas where participants reside, we used the only zip-codes that reflect their actual residence address without additional considerations such as occupation exposure due to absence of relevant data. Therefore, it did not reflect the case where the residence changed, and this method of assessing exposure levels using only zip-codes may not take into account many potential variables, such as occupational or indoor exposure and diversity of mobility. Third, we did not include data on exposure to PM_2.5_, which might have a greater impact on cSVD because of lack of relevant data. In addition, in the health screening process, data on the socioeconomic position were not available, which could act as a confounder of air pollutants. In addition, in the health screening process, data on the socioeconomic position were not available, which could act as a confounder of air pollutants. Finally, because the enrolled participants were limited to a single hospital, and this study is not representative of all Korean population. However, this study has a number of strengths. We assessed the estimated exposure to air pollutants on an individual basis to obtain information about the influence of air pollution on the cerebrovascular status of asymptomatic adults. We gathered extensive information on the potentially confounding factors for this study, and our analysis had a larger sample size than most prior studies did.

## Conclusions

Based on these analyses, this study identifies the association between annual exposure to PM_10_ and cSVD using brain MRI in Asian adults. Our findings suggest that exposure to air pollutants is a potential independent risk factor for cSVD in general adult population.

## Supplementary Information

Supplementary Information.

## Data Availability

Restrictions apply to the availability of data generated or analyzed during this study to preserve patient confidentiality or because they were used under license. The corresponding author will on request detail the restrictions and any conditions under which access to some data may be provided.
