# Supplementary material for: Annual exposure to PM10 is related to cerebral small vessel disease in general adult population
Source: Sci Rep. 2022 Nov 16;12:19693. doi: 10.1038/s41598-022-24326-y (PMC9668965; doi:10.1038/s41598-022-24326-y)
Supplement: Supplementary file 1 — Supplementary Information. [file 41598_2022_24326_MOESM1_ESM.docx]

**Annual Exposure to PM_10_ is Related to Cerebral Small Vessel Disease in General Adult Population**

Han-Yeong Jeong^1,2,*^, Hyun-Jin Kim^3,*^, Ki-Woong Nam^4^, Su-Min Jeong^5^, Hyuktae Kwon^5^, Jin-Ho Park^2,5,†^, Hyung-Min Kwon^2,4,†^

^1^Department of Neurology, Emergency Medical Center, Seoul National University Hospital, Seoul, Republic of Korea

^2^Seoul National University College of Medicine, Seoul, Republic of Korea

^3^National Cancer Control Institute, National Cancer Center, Goyang, Republic of Korea

^4^Department of Neurology, Seoul Metropolitan Government-Seoul National University Boramae Medical Center, Seoul, Republic of Korea

^5^Department of Family Medicine, Seoul National University Hospital, Seoul, Republic of Korea

Supplementary Table 1. The results for multicollinearity among variables

| **Variables** | **VIF (Variance Inflation Factor)** |
| --- | --- |
| Age | 1.140 |
| Sex | 2.193 |
| Body mass index | 1.086 |
| Hypertension | 1.126 |
| Systolic blood pressure | 3.715 |
| Diastolic blood pressure | 3.320 |
| Diabetes mellitus | 1.073 |
| Dyslipidemia | 1.038 |

Supplementary Table 2. Associations between independent variables with small vessel disease^a^

| **Characteristics**  **(n=3,257)** | **Volume of**  **white matter hyperintensity^b,c^** | | | **Silent lacunar infarct^b^** | | | | **Cerebral microbleeds^b^** | | | |
| --- | --- | --- | --- | --- | --- | --- | --- | --- | --- | --- | --- |
|  | **β** | **SE** | ***p*** | **Absent**  **(n=2,984)** | **Present**  **(n=273)** | **OR**  **(95% CI)** | ***p*** | **Absent**  **(n=3,122)** | **Present**  **(n=135)** | **OR**  **(95% CI)** | ***p*** |
| Age | 0.054 | 0.002 | <0.001 | 55.9  (9.3) | 63.2  (8.6) | 1.094 (1.078 – 1.110) | <0.001 | 56.3  (9.4) | 62.1  (9.0) | 1.070 (1.050 – 1.091) | <0.001 |
| Male | -0.026 | 0.040 | 0.517 | 1,605  (53.8) | 153  (56.0) | 0.913 (0.711 – 1.172) | 0.474 | 1,677  (53.7) | 81  (60.0) | 0.774 (0.544 – 1.100) | 0.152 |
| Height | -0.016 | 0.002 | <0.001 | 163.0  (8.8) | 161.9  (8.4) | 0.985 (0.971 – 0.999) | 0.042 | 162.9  (8.7) | 162.4  (9.5) | 0.993 (0.974 – 1.013) | 0.485 |
| Weight | -0.007 | 0.002 | <0.001 | 64.4  (11.1) | 64.1  (11.2) | 0.997 (0.986 – 1.008) | 0.610 | 64.4  (11.0) | 64.4  (12.2) | 1.000 (0.985 – 1.016) | 0.950 |
| BMI | 0.004 | 0.006 | 0.504 | 24.1  (3.1) | 24.3  (3.2) | 1.021 (0.981 – 1.063) | 0.304 | 24.2  (3.1) | 24.3  (3.3) | 1.016 (0.961 – 1.074) | 0.580 |
| Smoking | -0.058 | 0.026 | 0.026 |  |  |  |  |  |  |  |  |
| Never |  |  |  | 1,655  (55.5) | 147  (53.8) | Reference |  | 1,726  (55.3) | 76  (56.3) | Reference |  |
| Former-smokers |  |  |  | 795  (26.6) | 83  (30.4) | 1.175 (0.887 – 1.558) | 0.261 | 835  (26.7) | 43  (31.9) | 1.170 (0.797 – 1.715) | 0.423 |
| Current-smokers |  |  |  | 534  (17.9) | 43  (15.8) | 0.907 (0.637 – 1.291) | 0.587 | 561  (18.0) | 16  (11.9) | 0.648 (0.375 – 1.120) | 0.120 |
| Alcohol drinking | -0.094 | 0.021 | <0.001 |  |  |  |  |  |  |  |  |
| Never |  |  |  | 1,306  (43.8) | 127  (46.5) | Reference |  | 1,374  (44.0) | 59  (43.7) | Reference |  |
| Former-drinkers |  |  |  | 223  (7.5) | 23  (8.4) | 1.061 (0.665 – 1.691) | 0.805 | 234  (7.5) | 12  (8.9) | 1.194 (0.632 – 2.256) | 0.584 |
| Current- drinkers |  |  |  | 1,455  (48.8) | 123  (45.1) | 0.869 (0.671 – 1.126) | 0.289 | 1,514  (48.5) | 64  (47.4) | 0.984 (0.686 – 1.413) | 0.932 |
| Blood pressure |  |  |  |  |  |  |  |  |  |  |  |
| SBP | 0.011 | 0.001 | <0.001 | 125.7  (15.6) | 130.3  (16.0) | 1.019 (1.011 – 1.026) | <0.001 | 125.8  (15.6) | 130.8  (16.9) | 1.020 (1.009 – 1.030) | <0.001 |
| DBP | 0.008 | 0.002 | <0.001 | 75.8  (10.7) | 78.1  (11.1) | 1.019 (1.008 – 1.031) | 0.001 | 75.9  (10.7) | 77.6  (11.6) | 1.014 (0.999 – 1.031) | 0.074 |
| Hypertension | 0.436 | 0.040 | <0.001 | 1,097  (36.8) | 160  (58.6) | 2.436 (1.893 – 3.134) | <0.001 | 1,175  (37.6) | 82  (60.7) | 2.564 (1.801 – 3.649) | <0.001 |
| Diabetes mellitus | 0.444 | 0.055 | <0.001 | 429  (14.4) | 72  (26.4) | 2.133 (1.600 – 2.845) | <0.001 | 468  (15.0) | 33  (24.4) | 1.835 (1.224 – 2.750) | 0.003 |
| Dyslipidemia | 0.073 | 0.048 | 0.133 | 634  (21.2) | 68  (24.9) | 1.230 (0.922 – 1.640) | 0.160 | 671  (21.5) | 31  (23.0) | 1.089 (0.723 – 1.641) | 0.684 |

SE, standard error; OR, odds ratio; CI, confidence interval; BMI, body mass index; SBP, systolic blood pressure; DBP, diastolic blood pressure.

**^a^** Data are presented as mean (standard deviation) for continuous variables, or n (%) for categorical variables.

**^b^** Linear regression analysis was performed for volume of white matter hyperintensity, logistic regression analysis was performed for silent lacunar infarct and cerebral microbleeds.

**^c^** Volume of white matter hyperintensity was square root-transformed to achieve normality.

Supplementary Table 3. The difference in air pollutant exposure between cSVD (-) group and cSVD (+) group

|  | cSVD (-) group  (n=588) | cSVD (+) group  (n=2,669) | *p* |
| --- | --- | --- | --- |
| PM_10_, μg/m^3^ | 48.0±8.8 | 49.4±8.5 | <0.001 |
| NO_2_, ppb | 29.2±11.8 | 29.3±12.1 | 0.778 |
| SO_2_, ppb | 5.3±1.5 | 5.2±1.5 | 0.292 |
| CO, ppm | 0.57±0.14 | 0.56±0.14 | 0.763 |

Mean ± standard deviation

PM_10_, particulate matter ≤ 10 μm in diameter; NO_2_, nitrogen dioxide; SO_2_, sulfur dioxide; CO, carbon monoxide.

Supplementary Table 4. The annual averages of air pollutants during the study period in South Korea from the Annual Report of Air quality in Korea

| **Air pollutants** | **Year** | | | | | | | |
| --- | --- | --- | --- | --- | --- | --- | --- | --- |
|  | ‘06 | ‘07 | ‘08 | ‘09 | ‘10 | ‘11 | ‘12 | ‘13 |
| PM_10_, μg/m^3^ | 59 | 58 | 54 | 53 | 51 | 50 | 45 | 49 |
| NO_2_, ppb | 23 | 26 | 26 | 25 | 25 | 24 | 23 | 24 |
| SO_2_, ppb | 6 | 6 | 6 | 6 | 5 | 5 | 5 | 6 |
| CO, ppm | 0.6 | 0.6 | 0.6 | 0.5 | 0.5 | 0.5 | 0.5 | 0.5 |

Supplementary Table 5. Air pollutants (annual average concentrations) and their distributions

| **Air pollutants** | **Mean** | **SD** | **IQR** | **Percentile** | | | | | **Pearson’s correlation coefficients** | | | |
| --- | --- | --- | --- | --- | --- | --- | --- | --- | --- | --- | --- | --- |
|  |  |  |  | **10^th^** | **25^th^** | **50^th^** | **75^th^** | **90^th^** | **PM_10_** | **NO_2_** | **SO_2_** | **CO** |
| PM_10_, μg/m^3^ | 49.1 | 8.6 | 11.6 | 39.1 | 42.9 | 47.9 | 54.6 | 61.2 | 1 | 0.32**^a^** | 0.25**^a^** | 0.33**^a^** |
| NO_2_, ppb | 29.3 | 12.1 | 15.0 | 13.3 | 21.0 | 30.0 | 36.0 | 45.7 | - | 1 | 0.37**^a^** | 0.61**^a^** |
| SO_2_, ppb | 5.2 | 1.5 | 1.8 | 3.6 | 4.3 | 5.2 | 6.1 | 7.0 | - | - | 1 | 0.36**^a^** |
| CO, ppm | 0.57 | 0.14 | 0.17 | 0.39 | 0.47 | 0.57 | 0.64 | 0.74 | - | - | - | 1 |

SD, standard deviation; IQR, interquartile range; PM_10_, particulate matter ≤ 10 μm in diameter; NO_2_, nitrogen dioxide; SO_2_, sulfur dioxide; CO, carbon monoxide.

**^a^** *p*<0.001
